# Anatomical and Functional Effects of an Oral Supplementation of Bromelain and Curcugreen in Patients with Focal Diabetic Macular Edema

**DOI:** 10.3390/jcm12237318

**Published:** 2023-11-26

**Authors:** Adriano Carnevali, Sabrina Vaccaro, Massimiliano Borselli, Soufiane Bousyf, Luca Lamonica, Giorgio Randazzo, Giuseppe Giannaccare, Vincenzo Scorcia

**Affiliations:** 1 Department of Ophthalmology, University Magna Græcia of Catanzaro, 88100 Catanzaro, Italy; adrianocarnevali@unicz.it (A.C.); sabrina_vaccaro@libero.it (S.V.); mborselli93@gmail.com (M.B.); soufiane.bousyf@studenti.unicz.it (S.B.); lucalamonica24@gmail.com (L.L.); gioranda@libero.it (G.R.); vscorcia@unicz.it (V.S.); 2 Eye Clinic, Department of Surgical Sciences, University of Cagliari, 09124 Cagliari, Italy

**Keywords:** diabetic retinopathy, macula edema, optical coherence tomography, nutraceuticals, curcumin, bromelain

## Abstract

Diabetic retinopathy (DR) is one of the most severe diabetes-related complications, and macular edema stands as the primary contributor to the loss of central vision in individuals diagnosed with diabetes mellitus. The purpose of this study was to investigate the anatomical and functional effects of the oral administration of bromelain and curcugreen in patients controlled by therapy with non-proliferative DR presenting focal edema. Patients were enrolled and divided into two groups: group A (n = 18) received two tablets a day of bromelain and curcugreen (Retinil Forte^®^) orally, and group B (n = 15) underwent observation. The protocol included four visits: the screening visit (T0) and follow-up checks every 3 months up to 12 months (T3–T6–T9–T12). Best-corrected visual acuity (BCVA), central macular thickness (CMT) measured by optical coherence tomography (OCT) and vascular perfusion (VP) in superficial capillary plexus (SCP) and the deep capillary plexus (DCP) measured by optical coherence tomography angiography (OCTA) were analyzed. A mixed-design ANOVA was calculated to determine whether the change in BCVA, CMT, VP in SCP and DCP over time differed according to the consumption of Retinil Forte^®^. The results indicated that the interaction between time and treatment on the CMT and VP in DCP were significant, with F (4, 124) = 6.866 (*p* < 0.0001) and F (4, 124) = 3.263 (*p* = 0.0140), respectively. Conversely, the interaction between time and treatment was not significant on BCVA and VP in SCP with F (4, 124) = 1.121 (*p* = 0.3496) and F (4, 124) = 1.473 (*p* = 0.2146), respectively. In conclusion, our results suggest a protective role of the oral administration of bromelain and curcugreen in patients with DR and focal edema, in terms of the improvement of baseline CMT and VP in DCP over time.

## 1. Introduction

Diabetic retinopathy (DR) is one of the main complications of diabetes mellitus, with a prevalence of 22.27% [1]. It is the most common cause of preventable blindness in people of working age and it represents a serious public health issue [2]. Numerous mechanisms are involved in the intricate etiology of the disease. To explain it, a number of molecular theories have been proposed. Advanced glycation end products (AGEs), sorbitol accumulation, oxidative stress, protein kinase C activation, renin–angiotensin system upregulation and vascular endothelial growth factor (VEGF) are some of the pathways that have been implicated [3,4,5]. Increased levels of VEGF and other inflammatory agents cause the blood retinal barrier to be disrupted, increasing vascular leakage and decreasing fluid clearance, which results in the development of macular edema [6,7].

The most frequent cause of vision loss in people with diabetes mellitus is diabetic macular edema (DME), which is characterized as a retinal thickening that involves or approaches the macula’s center [8]. The typical course of DME is characterized by a gradual increase in retinal thickness until the central region of the macula is affected, leading to a decrease in visual acuity. DME seldom undergoes spontaneous resolution; rather, it commonly resolves due to alterations in various systemic risk factors, including glycemic control, hypertension, or hypercholesterolemia [9]. According to the current literature, intravitreal injections of anti-VEGF and dexamethasone are effective treatments for the majority of eyes with DME [10,11,12]. Despite the anatomical and visual improvement, complete edema resolution is not accomplished in a significant number of patients, or the retinal thickness is insufficient to warrant therapy with intravitreal injections [13]. From this perspective, nutraceutical treatment may seem like a reasonable alternative. In vitro and in vivo studies have revealed that some nutraceuticals, in particular, have anti-inflammatory and antioxidant properties that can prevent the molecular pathways that cause damage to the DR [14]. Retinil forte ^®^, a combination of bromelain and curcugreen, could find a reasonable use in the control of the inflammatory processes characterizing DR. Bromelain is a nutraceutical substance with anti-inflammatory and antioxidant properties. It is a sulfhydryl proteolytic enzyme present in pineapple plants, which carries out anti-inflammatory, cardioprotective, immunomodulatory, antioxidant and anti-tumor activities. Additionally, bromelain has anti-edema qualities [15,16]. Curcugreen, alternatively named BCM-95^®^, is composed of an 86% concentration of curcuminoid extract derived from turmeric, accompanied by 7–9% ar-turmerone oil [17]. In particular, curcumin, a component of turmeric, has been found to have therapeutic promise in delaying the course of DR due to its anti-inflammatory and antioxidant properties as well as its ability to block some nuclear transcription factors and vascular endothelial growth [18]. Furthermore, curcumin inhibits the enzymes cyclooxygenase (COX), lipoxygenase (LOX), and 5-hydroxy-eicosatetraenoic acid (5-HETE) to reduce inflammation [19,20]. Retinil forte ^®^ could be used in patients suffering from mild non-proliferative DR (NPDR) with focal edema, who do not meet the criteria for treatment with intravitreal injections.

With a high degree of accuracy and reproducibility, optical coherence tomography (OCT), a noninvasive, noncontact tool, provides cross-sectional, high-resolution images of the retina. It also provides a quantitative assessment of retinal thickness. Furthermore, using OCT, it is now possible to analyze macular morphology and central macular thickness (CMT) to look for any DR signs [21,22]. At the same time, the use of new imaging techniques such as optical coherence tomography angiography (OCTA) allow visualization in greater detail of the changes that take place at the level of retinal and choroidal vessels in patients with DR [23].

In light of this context, the aim of this study was to examine anatomical and functional effects of an oral administration of bromelain and curcugreen in patients presenting with NPDR with focal edema.

## 2. Materials and Methods

This is a prospective, non-randomized, monocentric study performed at the Magna Graecia University of Catanzaro between July 2022 and August 2023. The study was approved by the local ethics committee (Calabria Region Ethics Committee, Central Area Section, approval code CZ12106.). Before performing any procedure, patients read, understood and signed their informed consent. The study was carried out according to the regulations in force of the Helsinki Decree of 1964, and its later amendments.

Inclusion criteria were as follows: confirmed diagnosis of mild non-proliferative DR according to the International Clinical Diabetic Retinopathy (ICDR) and diabetic macular edema Severity Scale [24], naïve for any ocular treatment including intravitreal injection of anti-VEGF, age > 18 years, focal edema detected with OCT scan < 400 μm and diabetes well controlled by therapy (glycated hemoglobin values < 7 mg/dL and fast blood sugar FBS < 130 mg/dL). The exclusion criteria were other known retinal diseases associated with the development of macular edema, recent (within 3 months) ocular surgery, ischemic maculopathy, previous anti-VEGF-treatment, chronic degenerative systemic diseases (e.g., neoplasms) and the use of other natural dietary supplements for any reason. Moreover, patients considered eligible for intravitreal treatment by the treating ophthalmologist were not included in the study.

The sample method employed in this study was voluntary. The examiners were not masked and decided the treatment on clinical findings. Patients who satisfied study criteria were enrolled and divided into 2 groups: group A, which agreed to take two tablets of Retinil forte^®^ orally each day, containing bromelain (500 mg) and curcugreen (200 mg), and group B (the control group), which just underwent observation. The protocol included 4 visits: the screening visit (T0), and follow-up checks every 3 months up to 12 months (T3–T6–T9–T12). All patients underwent a complete visit examination, including Best-Corrected Visual Acuity (BCVA), testing 4 m logarithmic visual acuity chart with an Early Treatment Diabetic Retinopathy System (ETDRS) chart, intraocular pressure evaluation, slit lamp examination and fundus evaluation through indirect ophthalmoscopy; retinography performed using iCare EIDON and OCT.

OCT was performed using RTVue OCT (Optovue Inc., Fremont, CA, USA). CMT was obtained in retina map swabs measured by the Optovue algorithm and reviewed by an expert ophthalmologist (A.C.) to control the correct segmentation. OCTA was performed using XR Avanti AngioVue OCT-A (Optovue, Fremont, CA, USA), and quantitative data obtained from OCTA imaging included macular scans of 3 mm × 3 mm centered at the fovea. The instrument software (version 2017.1.0.151) automatically segmented OCT-A scans into en-face slabs: superficial capillary plexus (SCP) and the deep capillary plexus (DCP). Quantitative analyses of SCP and DCP were based on the default settings of the automated software algorithm of the AngioPlex (version 2017.1.0.151). The quantitative vascular measurement of SCP and DCP consisted of vascular perfusion (VP) (% of area occupied by vessels) in the whole zone for OCT-A 3 mm × 3 mm scan. The study assessed the anatomical and functional changes, specifically the reduction in CMT and improvement in VP and BCVA, during follow-up. These changes were then compared between the two groups.

### Statistical Methods

Statistical analysis was performed using Prism version 9.5.0 (GraphPad Software Inc., San Diego, CA, USA). Data were expressed as mean ± standard deviation (SD) if normally distributed, and otherwise as median values with interquartile range (IQR). The Anderson–Darling and Kolmogorov–Smirnov tests were applied to assess whether data were normally distributed. A Student’s *t*-test or Mann–Whitney U-test was applied to compare variables when appropriate. A mixed-design ANOVA was calculated to determine whether a change in CMT and BCVA over time occurred. A *p* value of less than 0.05 was considered statistically significant.

## 3. Results

A total of 33 patients (21 males, 12 females; mean age 64.30 ± 10.39 years) with a diagnosis of mild non-proliferative DR and focal edema were included in the study and divided in two groups: treatment (group A, n = 18) and observation (group B, n = 15). The main cohort data are summarized in Table 1.

At the baseline examination, there were no statistically significant differences between the two groups in terms of demographics, ocular parameters (BCVA, CMT, DCP, SCP), or in terms of glycated hemoglobin A1c levels.

**Figure 1:**
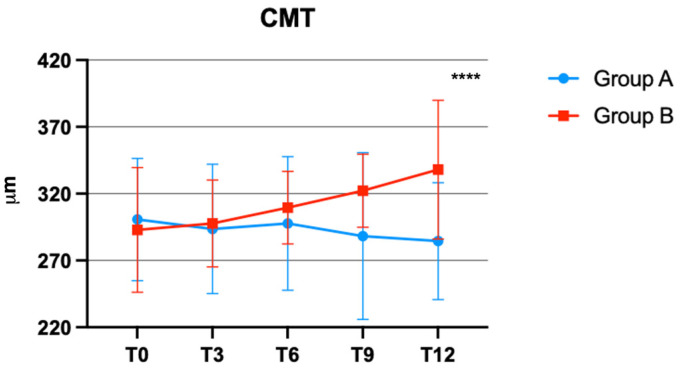
Central macular thickness (CMT) changes over time in groups A and B. **** *p* < 0.0001.

The results of the mixed-model ANOVA indicated that the interaction between time and treatment on the CMT was significant, with F (4, 124) = 6.866 (*p* < 0.0001). There was a reduction in CMT in patients of group A (Figure 1).

Specifically, during each follow-up time point, it was observed that group B showed a worsening in CMT dimension. Three patients from group B presented CMT > 400 µm at 12 months and subsequently were treated by the administration of an intravitreal injection of anti-VEGF.

**Figure 2:**
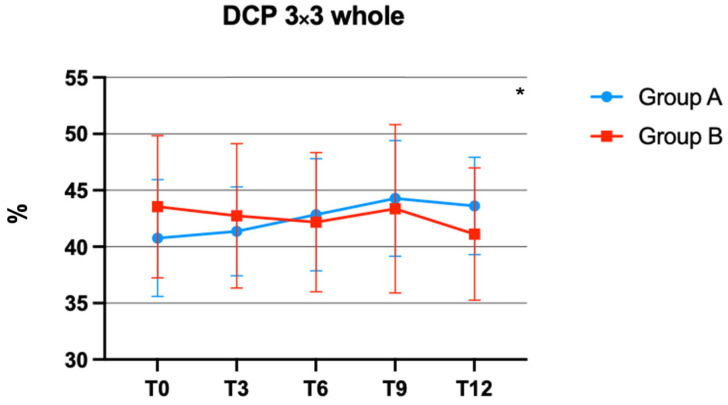
Deep capillary plexus (DCP) vascular perfusion (VP) changes over time in group A and B. * *p* < 0.05.

The interaction between time and treatment on DCP was significant, F (4, 124) = 3.263 (*p* = 0.0140), in different (Figure 2).

In particular, diabetic eyes of group A revealed a significantly increased VP when compared to control eyes of group B over time.

Conversely, the interaction between time and treatment was not significant in BCVA and SCP measured in a 3 × 3 scan, with F (4, 124) = 1.121 (*p* = 0.3496) and F (4, 124) = 1.473 (*p* = 0.2146), respectively. At the ophthalmic evaluation of 33 eyes analyzed, 5 were pseudophakic (group A: 3 eyes; group B: 2 eyes), 19 had an N2C3 cataract (group A: 10 eyes; group B: 9 eyes), 4 had an N3C2 cataract (group A: 2 eyes; group B: 2 eyes) and 5 had an N4C3 cataract (group A: 3 eyes; group B: 2 eyes). There were no documented adverse effects observed in group A, which received the oral administration of bromelain and curcugreen. Simultaneously, none of these individuals required the injection of intravitreal anti-VEGF therapy during the observation period.

In both groups, no statistically significant differences were detected with regard to glycated hemoglobin A1c values between baseline and T12 (group A: 6.72 ± 0.20% vs. 6.85 ± 0.33%, *p* = 0.1343; group B: 6.63 ± 0.23% vs. 6.70 ± 0.19%, *p* = 0.1906).

## 4. Discussion

DR continues to be the principal cause of legal blindness in working-age populations of industrialized countries, despite continuous advancements in diagnostic screening methods and available therapies [1,8]. Although the intravitreal injection of anti-VEGF and dexamethasone has demonstrated efficacy in treating eyes with DME, a considerable proportion of patients do not achieve the complete resolution of edema or present a retinal thickness that does not justify the use of an intravitreal injection as a treatment option [13].

The effects of curcumin on human retinal pigment epithelial cells (ARPE-19 cells) have been studied with respect to its protective properties against oxidative stress, induced cell death and hypoxia. Curcumin has been observed to cause a reduction in metabolic activity, which correlates with decreased cell proliferation, yet does not compromise cell survival. Accordingly, curcumin demonstrates protective effects against oxidative stress induced by H2O2 and also offers significant protection against cell death triggered by staurosporine [24].

In recent years, numerous beneficial effects of curcumin in the management of various ocular diseases have been demonstrated. Patients with recurrent anterior uveitis appeared to have better outcomes and reduced relapsing with oral curcumin in addition to traditional therapy [25]. Animal studies have shown that curcumin intake modulated inflammatory gene expression in rat retinas, exhibiting protective effects in age-related macular degeneration [26]. Moreover, it has been observed that for a beneficial effect of curcumin in humans, a lower dose is required compared to rats [27]. Additionally, in individuals with Central Serous Chorioretinopathy, the administration of oral curcumin supplements has demonstrated a notable enhancement in visual acuity due to its anti-inflammatory properties. This treatment could be regarded as a viable alternative for acute instances that do not resolve on their own and in chronic conditions [28].

Given that DR is a widespread and extensively researched condition, the antioxidant and anti-inflammatory effects of curcumin have shown effectiveness in reducing the molecular processes associated with this condition [14,15,16,17,18,19,20,21,22,23,24,25,26,27,28,29,30,31,32]. A study conducted in 2018 demonstrated the efficacy of a formulation containing curcumin in combination with standard therapies in patients suffering from DME. It was shown that this combined approach resulted in improved visual acuity and a reduction in DME [33]. At the same time, Guarino et al. documented the effectiveness of Curcuma longa and Boswellia serrata in individuals diagnosed with NPDR and who were treatment-naïve, and their findings indicated that these interventions were successful in preserving baseline CMT and best-corrected visual acuity BCVA values throughout the duration of the study [34].

In accordance with these findings, our study demonstrates a statistically significant decrease in CMT among patients suffering from mild non-proliferative DR with focal edema who received bromelain and curcugreen supplementation. In particular, this reduction, detected at the conclusion of the follow-up period, was found to be significantly different during each observation time.

Curcugreen is approximately 7–9 times more bioavailable than common curcumin, with a potency that is 700% greater than that of a normal turmeric 95% extract, and significant concentrations persist in the blood for up to 8 h after oral administration. The high bioavailability and rapid absorption promote anti-inflammatory activity and anti-edema effects at low doses [18].

Bromelain, another nutraceutical compound, exhibits several biological actions such as anti-inflammatory, antioxidant and anti-edema effects [19,20].

A considerable proportion of patients with mild non-proliferative DR and DME do not achieve the complete resolution of edema after intravitreal injections, or retinal thickness does not reach a level that justifies the use of this therapeutic intervention [13]. Our results suggest that Retinil forte^®^, a combination of curcugreen and bromelain, could be considered as an adjuvant therapy in cases of persistent focal edema in patients presenting with DR.

Nevertheless, the reduction of CMT was not accompanied by a statistically significant improvement in BCVA. However, we observed a trend towards BCVA reduction in patients not treated with curcugreen and bromelain and a substantial stability of visual acuity in subjects that received the treatments. Probably, this could be due to the small size of the study population. Moreover, the relatively poor vision in the groups could be explained by the presence of crystalline opacity, which was not an exclusion criterion.

In addition to the significant improvements in anatomic outcomes observed after the use of Retinil forte^®^, the results of this study also found a significant improvement in vascular perfusion in the DCP. A previous study demonstrated the efficacy of OCT-A in identifying early vascular changes in individuals diagnosed with type 1 diabetes mellitus (T1DM), in particular in analyzing the DCP, where diabetic eyes revealed a statistically significant decrease in VP compared to control eyes, suggesting that microvascular changes could precede the damage of DR [35].

Another study, conducted in 2022 by Chiosi et al., showed that a combination of curcumin and other nutraceutical substances may have a positive impact on the VP of the DCP in compensated type 2 diabetic patients with mild DME [36]. It has been demonstrated that there is an association between DCP reduction and DR progression [37].

At the same time, in our study, no statistical reduction in the VP of the SCP was observed, in agreement with the findings of a previous study [36]. The observed differences in the DCP parameters between the two groups may suggest that the oral supplementation with Retinil forte^®^ has a beneficial effect not only on CMT, but also on the wholeness of the VP of the DCP. The duration of the study spanned one year, providing an extensive follow-up period to evaluate the long-term effects of the therapy.

The inherent limitations of our study pertain to the very limited sample size of patients who were included. However, this factor did not provide a hindrance to the statistical analysis. Another significant limitation of this study was the absence of randomization, as well as the failure to account for the placebo effect by not administering a placebo pill to the control group.

In conclusion, our results suggest the protective role of the oral administration of bromelain and curcugreen in patients with mild NPDR and focal edema well-controlled by therapy, in terms of an improvement in baseline CMT and VP in DCP over time. The use of this association may represent a potential option to treat the considerable proportion of patients with mild NPDR that do not achieve complete resolution of edema or present retinal thickness that does not justify the use of intravitreal injection as a treatment option.

## Figures and Tables

**Table 1:** Baseline demographic, clinical ocular and systemic characteristics of study patients.

	Total n = 33	Group A n = 18	Group B n = 15	*p*
Age, mean ± SD, years	64.30 ± 10.39	68.56 ± 6.96	59.20 ± 11.69	0.2883
Male, N (%)	21 (63.63)	13 (72.22)	8 (53.33)	0.3005
BCVA, mean ± SD, ETDRS Letters	43.91 ± 7.34	43.06 ± 8.02	44.93 ± 6.55	0.4732
CMT, mean ± SD, μm	297 ± 45.61	300.7 ± 45.73	292.9 ± 46.69	0.6328
SCP 3 × 3 whole, mean ± SD, %	34.96 ± 3.69	35.00 ± 3.80	34.92 ± 3.68	0.9495
DCP 3 × 3 whole, mean ± SD, %	42.03 ± 5.80	40.77 ± 5.18	43.55 ± 6.30	0.1739
Glycated hemoglobin A1c, mean ± SD, %	6.68 ± 0.21	6.72 ± 0.20	6.63 ± 0.23	0.2510

BCVA = best-corrected visual acuity; CMT = central macular thickness; DCP = deep capillary plexus; SCP = superficial capillary plexus; SD = standard deviation.

## Data Availability

Data are contained within the article.
